# Angiopoietin-2 promotes osteogenic differentiation of thoracic ligamentum flavum cells via modulating the Notch signaling pathway

**DOI:** 10.1371/journal.pone.0209300

**Published:** 2018-12-17

**Authors:** Xiaoxi Yang, Zhongqiang Chen, Xiangyu Meng, Chuiguo Sun, Mengtao Li, Li Shu, Dongwei Fan, Tianqi Fan, Ann Y. Huang, Chi Zhang

**Affiliations:** 1 Department of Orthopedics, Peking University Third Hospital, Beijing, China; 2 Central Laboratory, Peking University International Hospital, Beijing, China; 3 Daobio, Inc. Dallas, Texas, United States of America; 4 Department of Orthopedics, Peking University International Hospital, Beijing, China; 5 Bone Research Laboratory, University of Texas Southwestern Medical Center, Dallas, Texas, United States of America; Augusta University, UNITED STATES

## Abstract

Thoracic ossification of the ligamentum flavum (TOLF) is heterotopic ossification of spinal ligaments, which may cause serious thoracic spinal canal stenosis and myelopathy. However, the underlying etiology remains inadequately understood. In this study, the ossification patterns of TOLF were analyzed by micro-computer tomography (micro-CT). The expression profile of genes associated with angiogenesis was analyzed in thoracic ligamentum flavum cells at sites of different patterns of ossification using RNA sequencing. Significant differences in the expression profile of several genes were identified from Gene Ontology (GO) analysis. Angiopoietin-2 (ANGPT2) was significantly up-regulated in primary thoracic ligamentum flavum cells during osteogenic differentiation. To address the effect of ANGPT2 on Notch signaling and osteogenesis, ANGPT2 stimulation increased the expression of Notch2 and osteogenic markers of primary thoracic ligamentum flavum cells of immature ossification, while inhibition of ANGPT2 exhibited opposite effect on Notch pathway and osteogenesis of cells of mature ossification. These findings provide the first evidence for positive regulation of ANGPT2 on osteogenic differentiation in human thoracic ligamentum flavum cells via modulating the Notch signaling pathway.

## Introduction

Thoracic ossification of the ligamentum flavum (TOLF) is a pathological heterotopic ossification that occupies the thoracic canal, which can cause severe thoracic myelopathy in East Asian population. Due to the progressive nature of the ossification and the refractoriness to conservative treatment, TOLF generally requires aggressive surgical intervention[1–4]. Several investigations suggested that potential contributing factors associated with TOLF, such as mechanical effects[5–7], inflammatory factors [8, 9]and genetic factors[10, 11], but the underlying mechanism of TOLF has not yet been clarified.

TOLF is a highly regulated development process, which can be described histologically based on endochondral ossification. Attention has recently been focused on the association between the effect of angiogenesis and endochondral ossification[12]. Evidences have been provided to indicate the involvement of angiogenic factors, such as vascular endothelial growth factor (VEGF)[13–15], angiopoietin (ANGPT) [16, 17] and hypoxia-inducible factor (HIF) [18, 19]in osteogenic differentiation. Other investigations also suggested the close correlation between the roles of angiogenic cytokines and the phases of inflammation in osteoblast differentiation[20, 21]. In the previous studies, VEGF/ANGPT-mediated angiogenesis has been revealed to be associated with the degenerative changes of ligamentum flavum hypertrophy [22–24]. However, it remains unknown whether angiogenic factors are involved in the process of TOLF.

Various signaling pathways have been associated with TOLF pathogenesis[25–27]. Among them, Notch signaling regulates angiogenic and osteogenic differentiation, and is increasingly recognized as a vital participant in skeletal development [28]. It has been reported that Notch pathway was involved in TOLF through promoting osteogenesis of ligamentum flavum cells[27, 29].

In this study, we evaluated the morphological characteristics of TOLF by micro-CT to investigate the ossification patterns. To explore the important role of angiogenesis in TOLF, RNA sequencing was utilized to identify several angiogenesis-related genes which are differently expressed between thoracic ligamentum fluvm cells of different patterns of ossification. According to the results of RNA-sequencing and Gene Ontology (GO) analysis, we investigated the effect of angiopoietin-2 (ANGPT2) on Notch signaling pathway and osteogenic differentiation in primary thoracic ligamentum flavum cells via ANGPT2 stimulation and knockdown. Our results implied that ANGPT2 positively regulate the osteogenic differentiation by affecting the Notch signaling pathway in human thoracic ligamentum flavum cells.

## Materials and methods

### Patient specimens

This study was approved by the Ethics Committee for Human Subjects of Peking University Third Hospital with the Declaration of Helsinki (PUTH-REC-SOP-06-3.0-A27, #2014003). The written consent was obtained. We investigated patients with TOLF who underwent decompressive laminectomy between August 2015 and May 2017 in our institution. All patients underwent posterior open decompressive laminectomy. During the surgery, the whole piece of ossified thoracic ligamentum flavum was carefully detached after resecting the lamina by ultrasonic bone curette.

### Micro-CT evaluation and measurements

The lamina resected with the whole ossified mass was examined to insure that the connection of the ossified ligamentum flavum was kept without damage. All the lamina specimens were scanned by micro-CT (Inveon, Siemens Medical Solutions, USA) with the scanning space resolution of 18μm (80kVP, 80μA, and 900ms exposure). Inveon Research Workplace (Version 3.0, Inveon) was utilized to manually draw around sites of ossified apophysis and calcification in the ligamentum flavum as region of interest (ROI) in each slicing image. These polygonal contours were then used to generate a 3-diemntional (3D) ROI for the subsequent analysis and calculation of the morphological parameters. In order to identify the ossification degree of the ligamentum flavum, the ossification bone volume/total volume (BV/TV), trabecular thickness (Tb.Th), trabecular number (Tb.N), and trabecular spacing (Tb.Sp) were calculated respectively.

### Cell culture and osteogenic differentiation

Ligaments were derived from patients during surgery and rinsed with phosphate-bufferedsaline (PBS). The ligaments collected were minced and digested using 0.25% trypsin (Gibco, Grand Island, NY, USA), followed by 250U/mL type I collagenase (Sigma-Aldrich, St.Louis, MO, USA). The specimen was then placed in100-mm culturing dishes containing Dulbecco’s Modified Eagle’s medium (DMEM; Gibco) supplemented with 10% fetal bovine serum (Gibco), 100U/mL penicillin G sodium and 100mg/mL streptomycin sulfate in a humidified atmosphere with 5% CO_2_ at 37°C. Passages 0 was used for subsequent experimentation when cell density reached 80%. To induce osteogenic differentiation, cells were cultured in osteogenic medium consisting of DMEM supplemented with 50μM ascorbic acid (Sigma-Aldrich), 10mM β-glycerophosphate (Sigma-Aldrich) and 10 nM dexamethasone (Sigma-Aldrich).

### RNA extraction and RNA sequencing

Total RNA was isolated using Trizol (Invitrogen, Carlsbad, CA). A total amount of 3 μg RNA per sample was used as input material for the RNA sample preparations. Sequencing libraries were generated using NEBNext Ultra RNA Library Prep Kit for Illumina (NEB, USA) following manufacturer’s recommendations and index codes were added to attribute sequences to each sample. The clustering of the index-coded samples was performed on a cBot Cluster Generation System using TruSeq PE Cluster Kit v3-cBot-HS (Illumia) according to the manufacturer’s instructions. After cluster generation, the library preparations were sequenced on an Illumina Hiseq platform and 125 bp/150 bp paired-end reads were generated. HTSeq v0.6.0 was used to count the reads numbers mapped to each gene. And then FPKM of each gene was calculated based on the length of the gene and reads count mapped to this gene.

### Differential expression analysis and GO enrichment analysis

Differential expression analysis was performed using the DESeq2 R package (For DESeq2 with biological replicates). The resulting *P*-values were adjusted using the Benjamini and Hochberg’s approach for controlling the false discovery rate. Genes with an adjusted *P*< 0.05 found by DESeq2 were assigned as differentially expressed; Prior to differential expression analysis, the read counts were adjusted by edgeR program package through one scaling normalized factor. Differential expression analysis was performed via the edgeR R package (For edgeR without biological replicates). Corrected *P*-value of 0.05 and absolute fold change of 2 were set as the threshold for significantly differential expression. GO enrichment analysis of differentially expressed genes (DEGs) was implemented by the cluster Profiler R package. GO terms with corrected *P*< 0.05 were considered significantly enriched by DEGs.

### Quantitative real-time polymerase chain reaction (qRT-PCR) analysis

Reverse transcription and qRT-PCR for ANGPT2 were performed using amiDETECTATrackmiRNA qRT-PCR Starter kit (RiboBio,Guangzhou,China). SYBR Green I was used for real-time PCR according to the manufacturer’s instructions (TaKaRa) with the Bio-Rad iQ5system (Bio-Rad). The relative gene expression levels were calculated using the 2^-ΔΔCt^ method. Reverse transcription and qPCR for the mRNA level of osteogenic markers was carried out as described previously [27]. All experiments were performed in triplicate. The following primer pairs were used: ANGPT2-Fw: 5’-AACTTTCGGAAGAGCATGGAC-3’; ANGPT2-Rv: 5’-CGAGTCATCGTATTCGAGCGG-3’;Notch1-Fw: 5’-CGCTGACGGAGTACAAGTG-3’; Notch1-Rv: 5’-GTAGGAGCCGACCTCGTTG-3’; Notch2-Fw: 5’-CCTTCCACTGTGAGTGTCTGA-3’; Notch2-Rv: 5’-AGGTAGCATCATTCTGGCAGG-3’.

### Western blot analysis

Total protein (50 μg) was separated in a Bis-Trispolyacryl amide gel and transferred onto a PVDF membrane (Millipore). The membrane was then incubated in 5% bovine serum albumin (BSA), and then incubated with a primaryantibody at 4°C overnight. Next, samples were incubated with IRDye800CWHRP-conjugated anti-IgG at room temperature for 1hour and visualized via chemiluminescence with an infrared laser scanning system (OdysseyLicor,Lincoln,NE,USA). The following primary rabbit-anti-human antibodies were used: anti-ANGPT2 (1:1000; Abcam); anti-Notch1 (1:500; Abcam); anti-Notch2 (1:500; Abcam); anti-Runx2 (1:1000;Abcam); anti-Sp7/Osterix (1:2000; Abcam); anti-ALP (1:2000; Abcam); anti-OCN (1:500; Abcam) and anti-GAPDH (1:2,500; Abcam).

### Alkaline phosphatase (ALP) activity assay

Cells were seeded in 6-well plates at the density of 1×10^5^ /well and cultured in osteogenic medium for 7 days. ALP activity was determined using an ALP activity staining kit (GENMED Scientifics, Shanghai, China).

### ANGPT2 stimulation/siRNA transfection

Hunman recombinant ANGPT2 (5μg/ml; R&D Systems, MN, USA) was used to treat primary thoracic ligamentum flavum cells of immature ossificationfor 48 hours during culture in osteogenic differentiation medium. Synthetic siRNA were purchased from RiboBio. SiRNA targeting ANGPT2 or Notch2 was transfected at a concentration of 250nM for mature ossification using Lipofectamine 2000 Transfection Reagent (Life Technologies, NY, USA), with non-targeting siRNA used as negative contral (NC).

### Statistical analysis

Data were presented as a mean±SD. The two-tailed unpaired Student’s *t*-tests were used for comparisons of two groups. The ANOVA multiple comparison test (SPSS 17.0) followed by Turkey *post hoc* test were used for comparisons of more than two groups. Each experiment was repeated at least three times. *P*<0.05 was considered to be statistically significant.

## Results

### Micro-CT scan and measurement of ossified ligamentum flavum in TOLF

**Fig 1:**
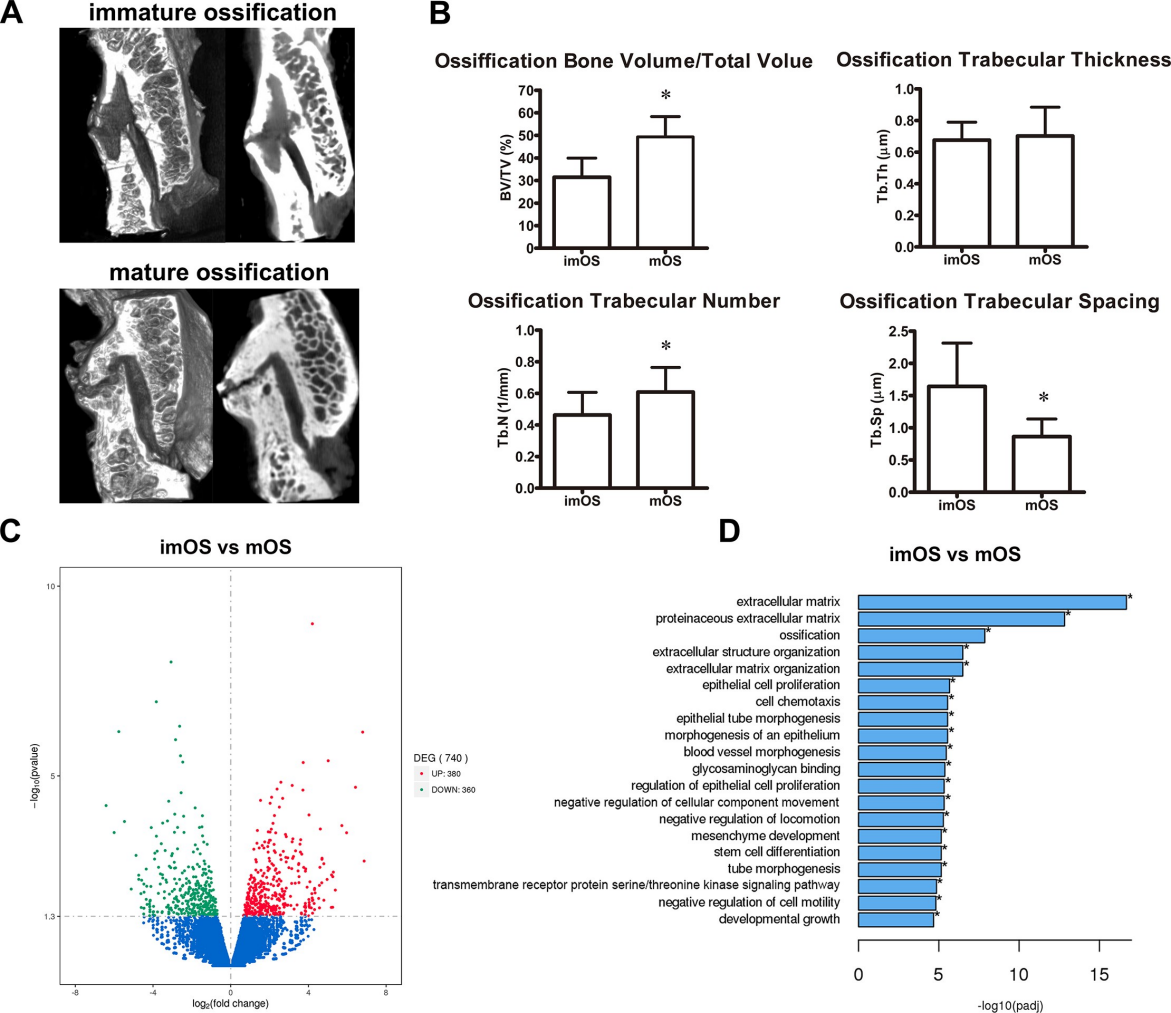
Comparison between immature ossification (imOS) and mature ossification (mOS) of TOLF. (A) Micro-CT images of immature ossification (imOS) and mature ossification (mOS) of TOLF; (B) morphological parameters; * *P*< 0.05 compared with imOS; (C) Volcano plots showing the DEGs among imOS and OS; (D) Differential GO terms distribution.

A total of 18 patients were enrolled in this study, including 9 men and 9 women with mean age of 55.50±9.53 years. There were 35 TOLF segments in total. As shown in **Fig 1A**, different ossification patterns of ligamentum flavum were observed. Two patterns of involvement were evident. Morphologically, among the 35 ossification segments, 16 were of immatureossification, 19 were of matureossification. The ossification degree was confirmed by quantitative measurements of morphological parameters in selected region of TOLF (**Fig 1B**).

### DEGs of ligamentum flavum via RNA-sequencing in TOLF patients

RNA-sequencing was utilized to investigate gene expression profile in different ossification patterns of the ligamentum flavum cells in TOLF. Raw sequencing data set was loaded as a supporting information file (S1 Dataset). There were totally 740 DEGs identified to be differently expressed, including 380 up-regulated DEGs and 360 down-regulated DEGs (**Fig 1C**). In order to identify the function of DEGs, we performed GO analysis. The classification of genes by biological process indicated that the DEGs were mainly involved inossification, stem cell differentiation, epithelial cell proliferation, extracellular matrix organization, blood vessel morphogenesis, etc **(Fig 1D)**. Among these DEGs, 42 osteogenesis-related factors were identified, SOX11, Runx2, Osterix (Osx), and secreted phosphoprotein 1 (SPP1) expressions were up-regulated, corresponding to the morphological data. Moreover, 35 angiogenesis-related factors were identified, including VEGFA, activin receptor like-protein 1 (ACVRL1), transforming growth factor beta 2 (TGFβ2), ANGPT2, ANGPT1, etc. Among them, ANGPT2 expression was up-regulated by 2.17-fold. Therefore, we focused on the effect of ANGPT2 on TOLF in the current study.

### Expression level of ANGPT2 in primary ligamentum flavum cells from TOLF patients

**Fig 2:**
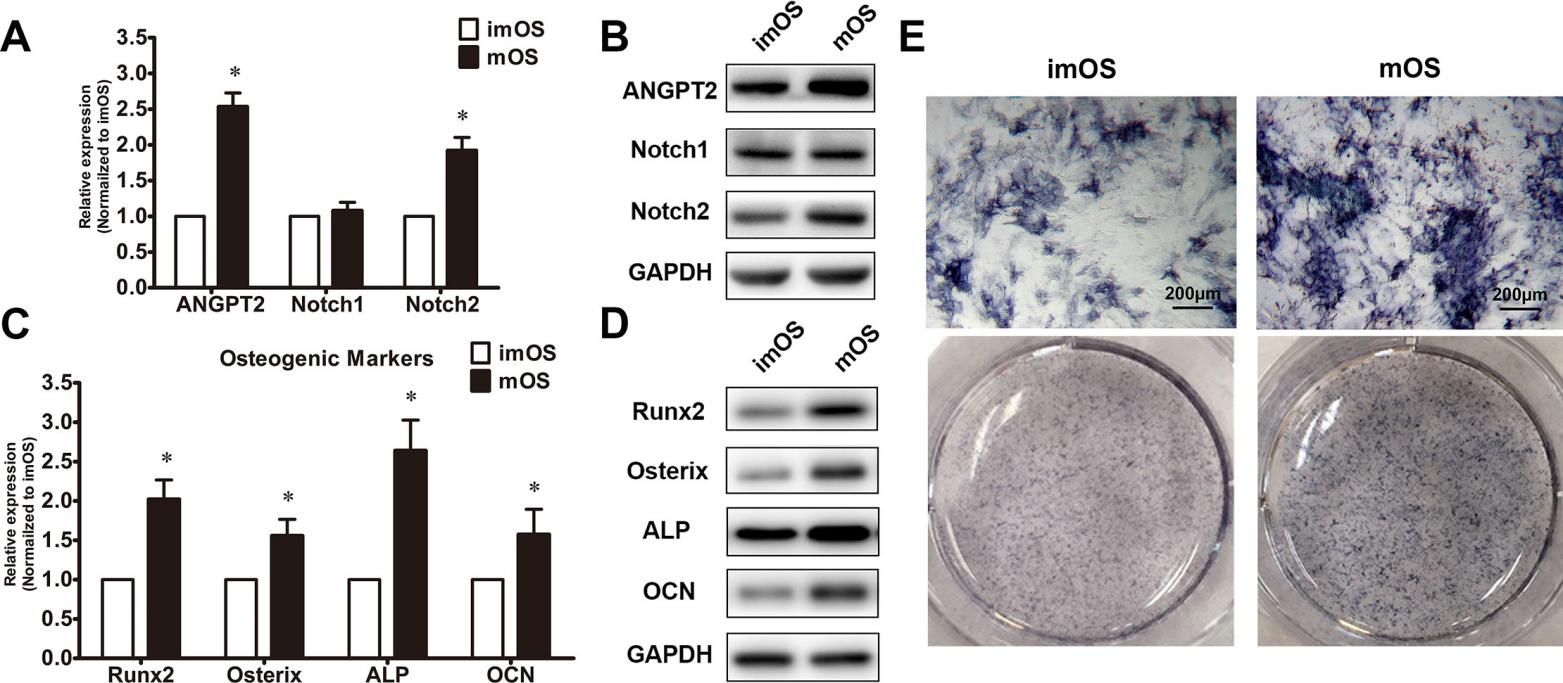
Gene activity comparison between immature ossification (imOS) and mature ossification (mOS) of TOLF. (A) Expression level of genes in primary thoracic ligamentum flavum cells. Expressions of ANGPT2, Notch1 and Notch2 were assessed via qRT-PCR.* *P*< 0.05 compared with imOS; (B) Protein levels in primary thoracic ligamentum flavum cells. Proteins of ANGPT2, Notch1 and Notch2 were analyzed via Western blot; (C) Osteogenic markergene expressionsin primary thoracic ligamentum flavum cells via qRT-PCR. * *P*< 0.05 compared with imOS; (D) Osteogenic marker protein levels in primary thoracic ligamentum flavum cells via Western blot; (E) ALP staining of ligamentum flavum cells from imOS and mOS patterns; scale bar represents 200 μm.

Cells were cultured in osteogenic medium for 7 days. Notch pathway activity and angiogenic and osteogenic abilities of primary cells were compared between immature and mature ossification sites in TOLF. Osteogenic induction resulted in increase in the expression of angiogenic genes ANGPT2, whereas cells of immature ossification exhibited insignificant increase. Specifically, Notch2 expression was significantly up-regulated in cells of mature ossification. Meanwhile, the levels of Runx2 and Osterix (Osx) were increased in a manner similar to ANGPT2 and Notch2 expression. The early osteogenic indicator alkaline phosphatase (ALP) and late indicator osteocalcin (OCN) were also significantly upregulated during ostegenesis. ALP activity was observed on day 7 (**Fig 2**).

### Effects of ANGTP2 on Notch signaling and osteogenic differentiation of primary ligamentum flavum cells from TOLF patients

**Fig 3:**
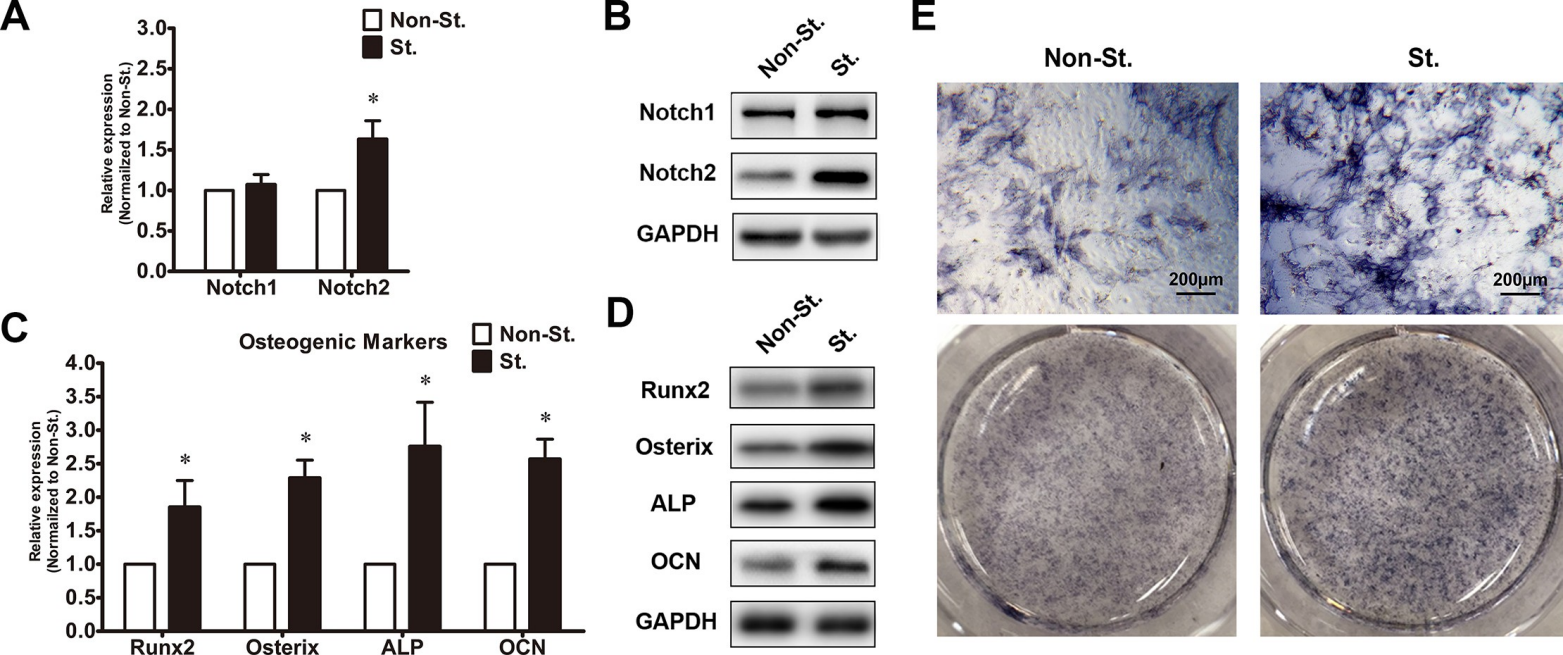
ANGTP2 stimulation enhances notch signaling and osteogenic differentiation of ligamentum flavum cells. (A) Effect of ANGPT2 on Notch1 and Notch2 expressions. Notch1 and Notch2 expressions were assessed via qRT-PCR in imOS ligamentum flavum cells with ANGPT2 stimulation (St.); * *P*< 0.05 compared with no stimulation group (Non-St.); (B) Effect of ANGPT2 on protein levels of Notch1 and Notch2 via Western blot; (C) Osteogenic marker expressions after ANGPT2 stimulation via qRT-PCR. * *P*< 0.05 compared with Non-St; (D) Effect of ANGPT2 on protein levels of osteogenic markers; (E) ALP staining following ANGPT2 stimulation; scale bar represents 200 μm.

**Fig 4:**
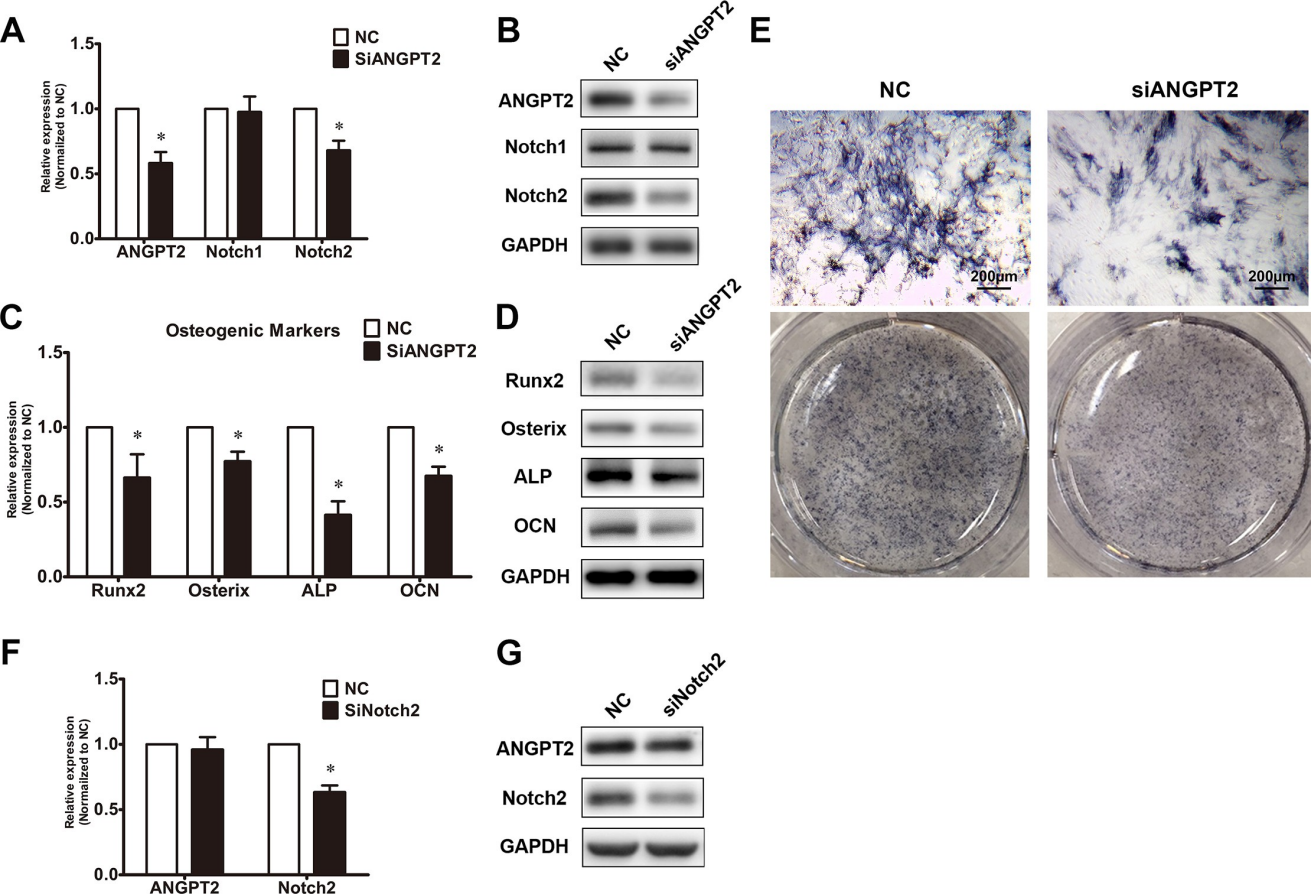
Effects of ANGTP2 knockdown on notch signaling and osteogenic differentiation of ligamentum flavum cells. (A) Effect of siANGPT2 on Notch1 and Notch2 expressions. ANGPT2, Notch1 and Notch2 expressions were assessed via qRT-PCR in mOS ligamentum flavum cells afterANGPT2 siRNA transfection (siANGPT2); * *P*< 0.05 compared with NC. (B) Effect of siANGPT2 on protein levels of Notch1 and Notch2 via Western blot; (C) Osteogenic marker expressions after siANGPT2 transfection via qRT-PCR.; * *P*< 0.05 compared with NC; (D) Effect of siANGPT2 on protein levels of osteogenic markers; (E) ALP staining following siANGPT2 transfection; scale bar represents 200μm; (F) Effect of Notch2 siRNA transfection (siNotch2) on ANGPT2 expression; * *P*< 0.05 compared with NC; (G) Effect of siNotch2 on ANGPT2 protein level.

The increased expression of ANGPT2 in TOLF led us to hypothesis that ANGTP2 may be involved in osteogenesis of thoracic ligamentum flavum cells. To investigate the effect of ANGPT2 on Notch signaling and osteognesis in TOLF, human recombinant ANGPT2 was used to treat primary cells of immature ossifcation during osteogenic differetiation for 48 hours. As shown in Fig 3, after ANGPT2 stimulation, the expressions of Notch2 and osteogenic markerswere significantly elevated compared to untreated control. Meanwhile, ANGTP2 stimulation showed an increase in activity of ALP staining (**Fig 3A–3E**). In contrast, ANGTP2 knockdown via siRNA transfection attenuated the expressions of Notch2 and the osteogenic differentiation of cells of mature ossification (**Fig 4A–4E**). Moreover, the ligamentum flavum cells transduced with Notch2 siRNA exhibited insignificant changes of ANGPT2 (**Fig 4F and 4G**). Our findings suggest that the ANGTP2 may induce the expression of osteogenic markers via modulating the Notch signaling pathway in primary thoracic ligamentum flavum cells.

## Discussion

TOLF is heterotopic ossification of spinal ligament, which can cause severe progressive thoracic spinal stenosis. The pathological process of TOLF involves the differentiation of fibroblasts into osteoblasts. The progression of TOLF is viewed as a process of endochondral ossification, followed by blood vessel invasion and replacement of the cartilaginous matrix with bone tissue. However, there has been no studies on the angiogenesis in TOLF. Thus, investigating angiogenesis and osteogensis of thoracic ligamentum flavum cells would likely lead to better understanding of the pathogenesis of TOLF. As far as we know, this is the first study to discover the involvement of the angiogenesis-related factor in TOLF.

In micro-CT, we first described the morphological characteristics of TOLF based on different ossification degrees. Two ossification patterns were discovered, including immature ossification and mature ossification. According to the different ossification patterns of TOLF, we examined the DEGs in the primary thoracic ligamentum flavum cells via RNA-sequencing. We found 740 DEGs, which were mainly involved in ossification, extracellular matrix organization, epithelial cell proliferation, blood vessel morphogenesis, etc. The insights we have acquired may be used for further research into the cause of TOLF.

Previous reports have suggested an important role of angiogenesis in bone formation, since the presence of a well-organized vascular plexus is vital to provide nurturing microenvironments to the newly forming tissue[30]. The results of GO analysis revealed that angiogenesis may play important role in the mechanisms of TOLF process. These angiogenesis-related factors include VEGFA, ANGPT2, ANGPT1, etc. The angiopoietins, ANGPT1 and ANGPT2 are increasingly recognized as essential angiogenic growth factors. ANGPT2 is an antagonist of ANGPT1, competitively binding to endothelial receptor TEK tyrosine kinase[31]. It was reported that ANGPT2 was found at sites of endochondral and intramembranous bone formation in the growing skeleton[32]. Even angiopoietin-like protein 2 (ANGPTL2), which is related to ANGPT2, has been also discovered to contribute to chondrocyte differentiation and subsequent endochondral ossification[16, 23]. The current study confirmed that ANGPT2 was up-regulated in the thoracic ligamentum flavum cells, corresponding to osteogenic markers, indicating the involvement of angiogenesis in TOLF. The combined results led us to further investigate the effect of ANGPT2 on osteoblast related genes.

In this study, our observations provide the first evidence that ANGPT2 promotes the osteogenic differentiation of thoracic ligamentum flavum cells. ANGPT2 stimulation induced the increased expression of the osteogenic differentiation of ligamentum flavum cells from TOLF patients; whereas the effect on osteogenesis was attenuated when ANGPT2 was knocked down by siRNA, thus indicating that the activation of ANGPT2 affected the osteogenic differentiation of thoracic ligamentum flavum cells. However, the underlying molecular mechanisms still need to be elucidated. It is known that endothelial Notch activity promotes angiogenesis and osteogenesis in bone[28]. Notch signaling pathways have been reported to mediate VEGF/ANGPT-2-induced angiogenesis and endothelial cell invasion in inflammatory arthritis[33]. Meanwhile, in our previous study, we found that Notch2 accelerated the osteogenic differentiation of flavum cells, which suggested that Notch signaling promotes osteogenic differentiation of thoracic ligamentum flavum cells[27]. In this study, ANGPT2 stimulation enhanced the expression of Notch2 and osteogenic differentiation, while ANGPT2 knock-down inhibited Notch signaling. In addition, down-regulation of Notch2 expression could not affect the expression of ANGPT2, thus suggesting that ANGPT2 induced osteogenic differentiation may be modulated through the Notch signaling pathway. Moreover, CCL5, a pro-inflammatory chemokine, has been reported to induce osteogenesis from MSCs to osteoblasts[34]. A recent study has shown that lactate activated human macrophages and stimulated the secretion of CCL5 by activation of Notch signaling [35]. In addition, CCL5wasproved to be up-regulated in the ANGPT2 protein-treated BMSCs[17]. Our data indicate that Notch signaling pathway is one mechanism for ANGPT2 to regulate osteogenic differentiation. This cannot rule out other possible mechanisms for ANGPT2’s effect on osteogenic differentiation. Therefore, further research is required to better comprehend the interactions between ANGPT2 and osteogenic transcription factors in ligamentum flavum cells.

In conclusion, RNA sequencing was utilized in this study to reveal differentiation gene profile of ligamentum flavum cells in TOLF. Our results demonstrated that the expression of ANGPT2 increased in the thoracic ligamentum flavum cells, which suggested that the activation of ANGPT2 enhanced the osteogenic differentiation in thoracic ligamentum flavum cells through the Notch signaling pathway.

## Supporting information

S1 Dataset Gene profile of ligamentum flavum cells in TOLF via RNA-sequencing. (XLS)
